# Bayesian Inference of Baseline Fertility and Treatment Effects via a Crop Yield-Fertility Model

**DOI:** 10.1371/journal.pone.0112785

**Published:** 2014-11-18

**Authors:** Hungyen Chen, Junko Yamagishi, Hirohisa Kishino

**Affiliations:** 1 Graduate School of Agricultural and Life Sciences, The University of Tokyo, Tokyo, Japan; 2 Institute for Sustainable Agro-ecosystem Services, The University of Tokyo, Tokyo, Japan; Chinese Academy of Sciences, China

## Abstract

To effectively manage soil fertility, knowledge is needed of how a crop uses nutrients from fertilizer applied to the soil. Soil quality is a combination of biological, chemical and physical properties and is hard to assess directly because of collective and multiple functional effects. In this paper, we focus on the application of these concepts to agriculture. We define the baseline fertility of soil as the level of fertility that a crop can acquire for growth from the soil. With this strict definition, we propose a new crop yield-fertility model that enables quantification of the process of improving baseline fertility and the effects of treatments solely from the time series of crop yields. The model was modified from Michaelis-Menten kinetics and measured the additional effects of the treatments given the baseline fertility. Using more than 30 years of experimental data, we used the Bayesian framework to estimate the improvements in baseline fertility and the effects of fertilizer and farmyard manure (FYM) on maize (*Zea mays*), barley (*Hordeum vulgare*), and soybean (*Glycine max*) yields. Fertilizer contributed the most to the barley yield and FYM contributed the most to the soybean yield among the three crops. The baseline fertility of the subsurface soil was very low for maize and barley prior to fertilization. In contrast, the baseline fertility in this soil approximated half-saturated fertility for the soybean crop. The long-term soil fertility was increased by adding FYM, but the effect of FYM addition was reduced by the addition of fertilizer. Our results provide evidence that long-term soil fertility under continuous farming was maintained, or increased, by the application of natural nutrients compared with the application of synthetic fertilizer.

## Introduction

Agricultural crop production is directly related to food supply, so agricultural soil productivity must be maintained. Balanced fertilization provides all the essential nutrients for crops to remain healthy and grow productively [1]–[3]. In a world facing increasing population pressure [4], our highest priority must be to increase crop productivity to ensure food security [2], [5]–[7]. In this context, there has been increasing concern about the long-term productivity of soils on a global scale [8]–[11].

The relationship between crop yield and soil fertility under different fertilization regimes has been studied for decades. Aref and Wander [12] investigated the long-term trends of corn yield and soil organic matter in different crop sequences and soil fertility treatments. Merick and Németh [13] used results from 60-year field experiments to provide information on the relationship between fertilization and yields of rye and potato. Hallin et al. [14] investigated the relationship between microbial communities and total crop yield and nitrogen content in the crop in a 50-year-old fertilization experiment. Fan et al. [15] studied the trends in grain yields and soil organic carbon (SOC) in a 26-year dryland fertilization trial. By carrying out a 27-year experiment with various fertilization treatments in a rotation cropping system with wheat and maize in a red soil, Zhang et al. [16] investigated trends in SOC, soil nitrogen, and grain yield.

Many researchers have suggested that soil fertility under continuous farming is maintained, or increased, by the application of farmyard manure (FYM) compared with the application of synthetic fertilizer [2], [11], [17]–[19]. To ensure gains in crop productivity, it is necessary to maintain and even improve the soil fertility of a field under continuous farming practice [20]–[23]. In some cases, soils do not contain sufficient amounts of the essential nutrients required for rapid crop growth and high productivity [2], [7], [24]. As a result, supplemental nutrients, applied as fertilizers, manure or compost, are needed [25]–[27]. Many studies have analyzed the effects of manure [28]–[30], chemical fertilizer [31], [32], and both [33], [34] on soil fertility and crop yield.

Previous models have predicted the crop-yield response to fertilizer application [19] and climate change [35] and have greatly contributed to the software used in agricultural systems research [36], [37]. Myers [38] used a static model to estimate the nitrogen fertilizer requirements of cereal crops. Deng et al. [39] proposed a theoretical framework that predicts the optimum planting density and maximal yield for an annual crop plant. Cong et al. [40] indicated that the CENTURY model [41] can simulate fertilization effects on SOC dynamics under different climate and soil conditions.

Soil quality is a complex combination of biological, chemical, and physical properties. In this paper, we focus on the application of these concepts to agriculture and define the baseline fertility as the level of soil fertility that crops utilize for their growth. Therefore, our soil quality is an interaction of the above properties and the crop activities. With this strict definition of baseline fertility, we propose a new crop yield-fertility model that enables quantification of the process of improving baseline fertility and the effects of treatments solely from the time series of crop yields. The model was modified from Michaelis-Menten kinetics and measured the additional effects of the treatments given the baseline fertility. Using more than 30 years of experimental data of maize (*Zea mays*), barley (*Hordeum vulgare*), and soybean (*Glycine max*) yield, we estimated in the Bayesian framework the improvements in baseline fertility and the effects of fertilizer and farmyard manure (FYM). We compared the efficiency of separate applications of fertilizer or FYM to the three crops (i.e., maize, barley and soybean) using estimated model parameters. The temporal variations in the baseline fertility of each crop were estimated and compared for six treatments and two soil types.

## Materials and Methods

### Field experiments

The long-term fertilizer experiment was conducted between 1980 and 2010 at the University Farm at the Institute for Sustainable Agro-ecosystem Services at the University of Tokyo, Nishitokyo, Tokyo, Japan (35°43′ N latitude and 139°32′ E longitude and an altitude of 53 m above mean sea level). The field site was located in the Kantō Plain, where the soil is covered with pyroclastic material from volcanoes that surrounded the western Kantō region 126,000 years ago. The soil parent material is Tachikawa loam. The surface soil is a black-colored fertile andosol containing a high percentage of humus and the subsurface soil (25 cm below the surface) is red-colored barren clay. Andosols are soils found in volcanic areas formed in volcanic tephra. Andosols have a different composition from chernozems and are not commonly found outside the Pacific “ring of fire”. At the beginning of the experiment, the percent nitrogen was 2.23 g/kg in the surface soil and 0.97 g/kg in the subsurface soil. A 2-year crop rotation of maize-barley-soybean-barley was maintained throughout the experimental period and the crop yields were measured. The maize crop was sown at the beginning of July and harvested at the end of September every second year from 1980 onwards. The barley crop was sown in the first half of November and harvested either at the end of May or at the beginning of June of the next year each year from 1980 onwards. The soybean crop was sown at the end of June and harvested in the second half of October every second year from 1981 onwards. The seeds of the three crops were ridge sown using a seeding machine. For maize and soybean, one seed was sown in each hole, and the widths between the ridges and stocks were 71 cm and 23 cm, and 71 cm and 17.5 cm, respectively. For barley, row seeding was applied using a width of 17.8 cm between the ridges.

Six treatment plots containing the NPK fertilizer applications and two levels of farmyard manure (FYM) combined with compost were established in both of the fields with the surface soil (64 m^2^ per plot, 8×8 m) and the subsurface soil (56 m^2^ per plot, 7×8 m) and were replicated four times. The percent nitrogen, phosphorus, and potassium in the fertilizer treatments were N:P:K = 12:7.9:13.3 for maize and barley and 3:4.4:8.3 for soybean. Fertilizer was added to the soil at a rate of 1 t ha^−1^ for all crops. The FYM comprised wheat straw and cow dung. The percent nitrogen in the FYM ranged from 0.2–0.4. FYM was added to the plots at two levels, i.e., 20 and 60 t ha^−1^. The FYM treatments were added to the soil twice a year from 1980 to 1990 before the crops were sown and once a year (before the barley was sown) from 1991 onwards. After the barley harvest in 2008, fertilizer and FYM applications were discontinued for all treatments. Phosphate fertilizer was added simultaneously with the NPK fertilizer and FYM at a rate of 2 t ha^−1^ after the soybean harvest from 1980 until 2001. All aboveground components were removed from the field during harvesting. The dead foliage, below-ground components, and remnant stem sections (i.e., 15 cm for maize and 10 cm for barley and soybean) remained in the field. The harvested material from the six treatments for both the surface and subsurface soils were weighed after drying at 80°C. The dry weight (g m^-2^) of the aboveground maize components, the barley spike, and the soybean seed were measured.

### Testing significance of yield differences in different soils

The crop yields of the surface and subsurface soils without the addition of fertilizer or farmyard manure (FYM) reflect the yield differences that occurred purely as a result of the soil. The crop yields observed for the subsurface soil were 39±25%, 49±20%, and 19±31% (±SD) lower than the yields observed for the surface soil for maize (Fig. S1A), barley (Fig. S1B), and soybean (Fig. S1C), respectively. There was a difference in yield between the two types of soils but it was only significant for barley (t = −3.69, *P*<0.001; for maize, t = −1.58, *P* = 0.13 and for soybean, t = −0.94, *P* = 0.35). Although the crops were planted in the same fields with the same soil, they obtained and used nutrients from the soil in a manner unique to each crop.

The maize and soybean yields did not differ significantly (*P*>0.2) between the surface and subsurface soils when fertilizer or FYM or a combination of fertilizer and FYM were applied (Fig. S1A and C for maize and soybean, respectively). The barley yields differed significantly between the surface and subsurface soils when both fertilizer and FYM (t = 2.27, *P*<0.05) or FYM alone (t = 2.18, *P*<0.05) were added, but did not differ significantly when only fertilizer was added (t = −0.63, *P* = 0.52) (Fig. S1B). The difference in yields between the surface and subsurface soils was reduced when either fertilizer or FYM was added to the soil. The application of fertilizer and FYM improved the yields for both the surface and subsurface soils and reduced the yield differences between the soil types. This finding indicates that productivity can be increased to an average level by fertilization even in a field with barren soil. While this may have been prior knowledge, the manner in which fertilizer and FYM contribute nutrients to the soil and how the crops make use of the nutrients obtained from these sources remains unclear.

### Testing significance of yearly variance

The maize and soybean yields increased significantly (*P*<0.05) for both soil types over the 30-year period. The barley yield increased significantly in the subsurface soil (*P*<0.05) but not in the surface soil (*P* = 0.15). The soils were fertilized by crop residues (i.e., roots, fallen leaves, and stem sections (15 cm above ground for maize and 10 cm for barley and soybean)) that remained in and on the soil after harvesting. Organic matter accumulated in the soil over time, providing a long-term, slow-release source of nitrogen, phosphorus, sulfur, and other important nutrients for crop growth, which resulted in increased soil productivity.

### Testing significance of the treatment effects

We used a nested analysis of variance to compare the yields of the three crops in the surface and subsurface soils in response to the different treatments. The surface soil produced significantly greater barley crop yields compared with the subsurface soil (Fig. S1B, F = 14.68, *P*<0.001). Maize and soybean yields (Fig. S1A, F = 2.91, *P* = 0.09 and Fig. S1C, F = 2.19, *P* = 0.14, respectively) did not differ significantly between the two soil types. The interaction effects of fertilizer and FYM were significant for maize (F = 3.32, *P*<0.05) and barley (F = 10.71, *P*<0.001), but not for soybean (F = 0.69, *P* = 0.60). The soybean yield did not differ significantly among the treatments (*P*>0.1 for both fertilizer and FYM).

### The derivation of the crop yield-fertility model

**Figure 1:**
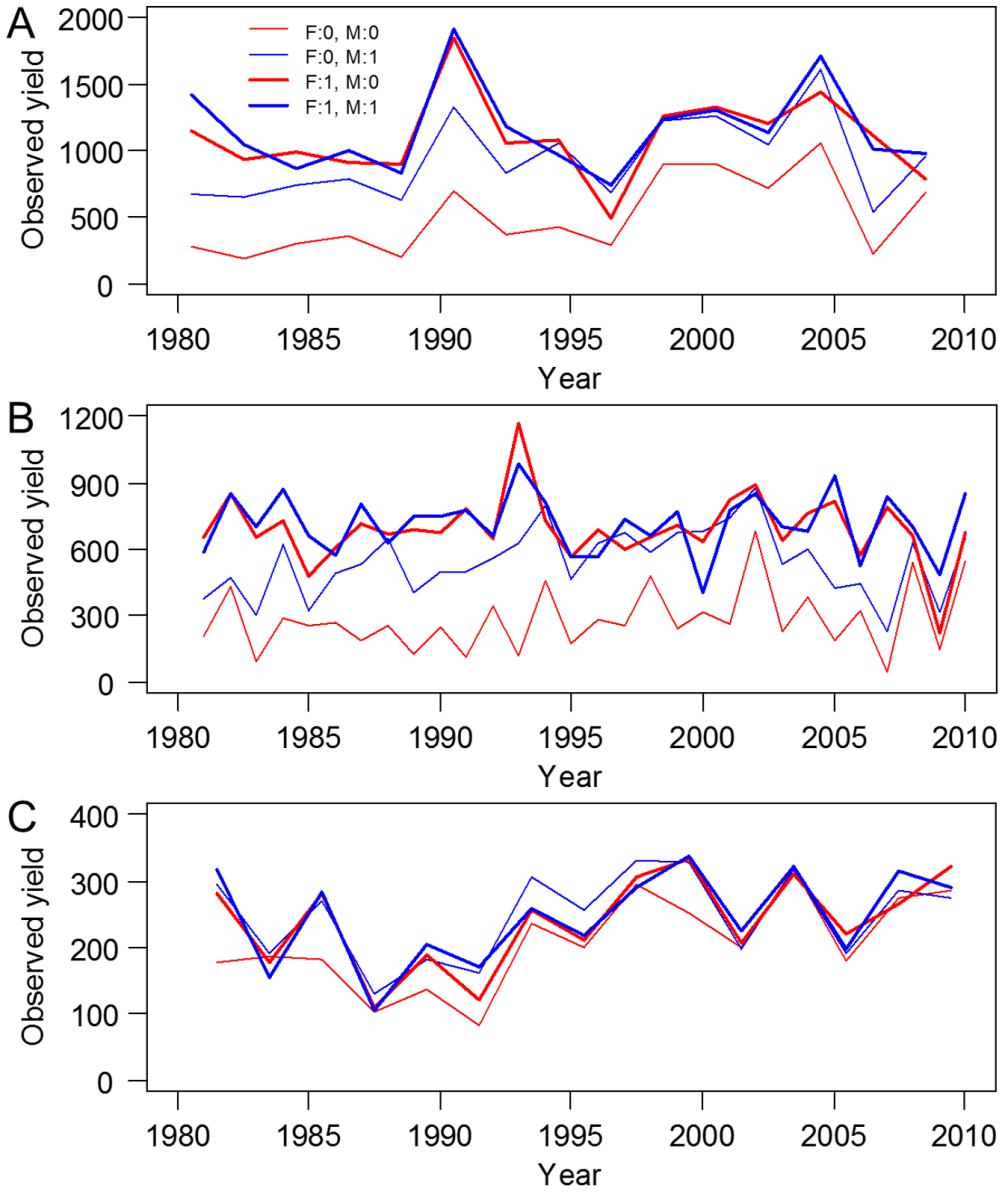
Temporal variations in observed crop yield (g m^−2^) for four treatments in the surface soil. (A) Maize; (B) Barley; (C) Soybean; F, level of fertilizer; M, level of farmyard manure. See Fig. S1 for all treatments in surface and subsurface soils.

Our model is based on the general pattern of the observed crop yield. The temporal variation in maize yield in response to four treatments applied to the surface soil over 30 years is shown in Fig. 1A. There were clear differences in the yield between the four treatments prior to 1982 (the first two data points). The response of the crop yield descended in the following order: the addition of NPK fertilizer and FYM (F:1, M:1), the addition of NPK fertilizer only (F:1, M:0), the addition of FYM only (F:0, M:1), and no nutrient application (F:0, M:0). The yield differences between the treatments receiving NPK fertilizer and FYM (F:1, M:1) and NPK fertilizer only (F:1, M:0) could not be detected from 1984 onwards. The effect of FYM could no longer be detected once the fertilizer application was continued. From 1994, similar crop yields were observed in the treatments with fertilizer, FYM or a combined application of fertilizer and FYM. In the last year of the study, there were negligible differences between treatments because fertilizer and FYM applications were discontinued in 2008. The soybean data are presented in Fig. 1C. The pattern of the soybean yield differed from maize. There were almost no differences in crop yield among the three treatments that received fertilizer, FYM or a combined application of fertilizer and FYM in the first and later years. The differences in crop yield between the treatments with and without fertilizer were negligible from 1995 onwards. The inherent soil fertility may be sufficient to grow soybean without the addition of fertilizer. The pattern of barley yield (Fig. 1B) was similar to maize but was not as well defined.

**Figure 2:**
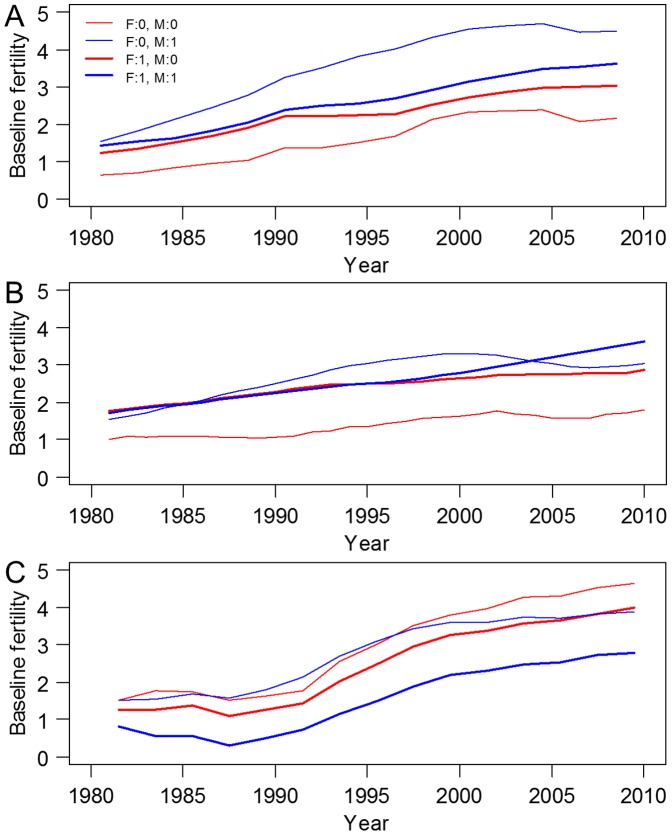
Crop yield-fertility curves for (A) Maize, (B) Barley, and (C) Soybean. The value of 1 for fertility is the fertility without the addition of fertilizer or FYM in the initial year for the subsurface soil. F, level of fertilizer. See Figs. S2A, S3A, and S4A for the band that corresponds to the standard deviation of the curves.

Our model can predict crop yields given the total fertility level of the soil. The model can be used to describe the variations in crop yield and the effect of different fertilizer treatments over different years for each crop using the estimated soil fertility. The crop yield-fertility curves for maize, barley, and soybean are presented in Fig. 2A, B, and C, respectively. Soil fertility was increased by the application of fertilizer over a 20-year period. Furthermore, the effect of fertilizer decreased over time for all three crops from 1980–2000.

### The crop yield-fertility model

We developed a simple mathematical model to estimate the soil fertility level and to quantify the contributions of fertilizer and FYM to the improvements in crop yields. The model was modified from Michaelis-Menten kinetics [42]:
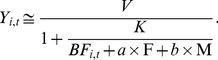
(1)


We did not apply the model to describe the kinetics. Rather, we used a form of the model to interpret the effects of baseline fertility, fertilizer, and FYM on the crop yield and the extent of saturation of additional inputs. The model related crop yields (Y_i,t_) to the total fertility (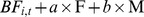) of the soil for treatment *i* at time *t* (Fig. 2). The value of the baseline fertility (*BF*) for the subsurface soil without the addition of fertilizer or FYM in the first year was normalized by setting this value to 1. *V* is the maximum yield in response to the maximum fertilizer input; *K* is the fertility level before fertilization that is required to produce half of the maximum yield, *V*. A large *K* indicates that more nutrients need to be added to the soil for the crop to grow, so *K* can infer the sterility of the soil prior to fertilization. 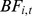 is the baseline fertility of the crop for treatment *i* at time *t* and is assumed to vary gradually over time; F is the level of fertilizer application (0 and 1), M is the level of FYM application (0, 1/3, and 1), and *a* and *b* represent the contributions of F and M relative to the baseline fertility, respectively. Hereafter, we refer to *V*, *K*, *a*, and *b* as the maximum yield, half-saturated fertility, fertilizer contribution, and FYM, respectively. A Bayesian framework was adopted for parameter estimation assuming a gradual change in the baseline fertility.

### The likelihood and priors

The likelihood of the yield for treatment *i* at time *t* followed a normal distribution with the mean 
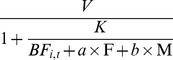
 and the variance *δ*:
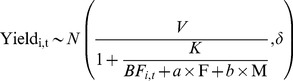
(2)


This value was normalized by setting the *BF* of the subsurface soil without the addition of fertilizer or FYM in the first year to 1. The priors of the *BF* for the other treatments in the first year followed a gamma distribution with a shape parameter of 1 and a scale parameter of 1. The smoothness priors of the *BF* from the second year followed a normal distribution with the mean equal to the value of the *BF* in the preceding year and the variance *τ*:
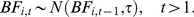
(3)


The inverse of *δ* followed a gamma distribution with a shape parameter of 0.1 and a scale parameter of 10. The inverse of *τ* followed a gamma distribution with a shape parameter of 0.1 and a scale parameter of 10. The prior of *V* followed a normal distribution with a mean of 0 and a standard deviation of 1000. The prior of *K* followed a gamma distribution with a shape parameter of 0.1 and a scale parameter of 10. The priors of *a* and *b* followed a gamma distribution with a shape parameter of 1 and a scale parameter of 1. The priors of the estimates were designed to be as non-informative as possible within a realistic range of the parameter values. All calculations and data analyses were performed using R v2.13.2 [43]. The raw data is available as (Data S1).

## Results

### Increasing trend of soil fertility

**Figure 3:**
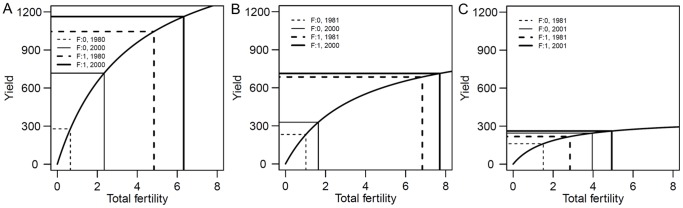
Temporal variations in the baseline fertility for four treatments in the surface soil. (A) Maize; (B) Barley; (C) Soybean; F, level of fertilizer; M, level of farmyard manure. See Fig. S5 for all treatments in surface and subsurface soils.

The baseline fertility of the surface and subsurface soils in all treatments increased over the 30-year period for all three crops (Figs. 3, S5–6). The baseline fertility of the subsurface soil was very low for maize and barley but was close to half-saturated for soybean when fertilization was initiated.

The treatment differences in the baseline fertility (i.e., maximum minus minimum) were increased by continuous cropping over the experimental period. The difference in the baseline fertility between the treatments was 0.89 in 1980 and 3.24 in 2008 for maize; for barley, it was 1.20 in 1980 and 2.54 in 2010, and for soybean, it was 0.96 in 1980 and 3.19 in 2009.

We used a 2×2 factorial analysis of variance to compare the baseline fertility of the three crops in response to the different treatments. The interaction effects of fertilizer and FYM were significant for maize (F = 15.09, *P*<0.001) and barley (F = 50.67, *P*<0.001), but not for soybean (F = 1.77, *P* = 0.19). The baseline fertility of soybean differed significantly for fertilizer (F = 10.26, *P*<0.01) but not for FYM (F = 4.00, *P* = 0.05).

### The effects of fertilizer and FYM

The Bayesian estimates of the model for the three crops are shown in Table 1. The traces of the MCMC (Markov Chain Monte Carlo) samples show the well-mixing and convergence to the posterior distributions (Fig. S7). We compared the mean values of the parameters for the three crops. The maize crop had the highest half-saturated fertility level and maximum yield. The soybean crop had the lowest half-saturated fertility level and maximum yield. For maize, the size of the fertilizer contribution was almost equal to the size of the half-saturated fertility, and FYM contribution was 81% smaller than the half-saturated fertility. For barley, the fertilizer contribution was 40% higher than the half-saturated fertility and the FYM contribution was 65% lower than the half-saturated fertility. This indicates that the amount of fertilizer applied in the experiment was 40% more than the fertility level at which the barley yield was half of the maximum yield, but the amount of FYM applied was 65% less than required. For soybean, the values of the fertilizer and FYM contributions were similar, i.e., 16 and 13%, respectively, and were lower than the half-saturated fertility.

**Table 1:** The posterior mean and standard deviation (SD) of the Bayesian estimates.

Crop	*V* (g m^−2^)		*K*		*A*		*B*	
	Mean	SD	Mean	SD	Mean	SD	Mean	SD
Maize	1833.01	316.56	3.65	1.68	3.61	1.27	0.68	0.53
Barley	1042.87	134.97	3.56	1.18	5.07	1.44	1.25	0.63
Soybean	360.17	65.54	1.87	1.01	1.58	0.58	1.62	0.94

*V*, maximum yield; *K*, half-saturated fertility; *a*, fertilizer contribution; *b*, FYM contribution.

In contrast with maize and barley, the value of the FYM contribution was higher than the fertilizer contribution for the soybean crop. The application rate of fertilizer and FYM approximated the fertility level at which the soybean yield was half of the maximum yield. The half-saturated fertility level for maize was 3.65, and the half-saturated fertility levels for barley and soybean were 2 and 49% lower, respectively. The fertilizer contribution for barley was 5.07, and the fertilizer contributions for maize and soybean were 29 and 69% lower, respectively. The FYM contribution for soybean was 1.62, and the FYM contributions for maize and barley were 58 and 23% lower, respectively. The model estimates revealed how the different crops used the nutrients from fertilizer and FYM applications to the soil. Maize needed the highest level of fertility to reach half of the maximum yield (i.e., the largest half-saturated fertility) compared with barley and soybean. Fertilizer contributed the maximum amount to the barley yield, and the FYM contributed the maximum amount to the soybean yield.

The total fertility levels among the treatments for both surface and subsurface soils during the 30-year period ranged from 0.54 to 7.22 for maize (Fig. S2A), 0.41 to 9.78 for barley (Fig. S3A), and 0.90 to 5.56 for soybean (Fig. S4A). Barley may be the most efficient crop in terms of fertilizer use among the three crops. The maximum total fertility obtained for soybean was 2.97× the half-saturated fertility, which is larger than the values obtained for maize (1.98) and barley (2.75).

Using the total fertility range, we estimated the predicted range in crop yield using the crop yield-fertility model for each crop. The predicted crop yields ranged from 236.24 to 1217.51 g m^−2^ for maize, 107.70 to 764.56 g m^−2^ for barley, and 117.02 to 269.52 g m^−2^ for soybean. Because of fluctuations in crop yield that could not be described by the model, the predicted yields covered 49, 57, and 42% of the observed yield ranges for maize (35 and 2023 g m^−2^), barley (14 and 1168 g m^−2^), and soybean (16 and 381 g m^−2^), respectively.

### The decreasing effect of fertilization over time

**Figure 4:**
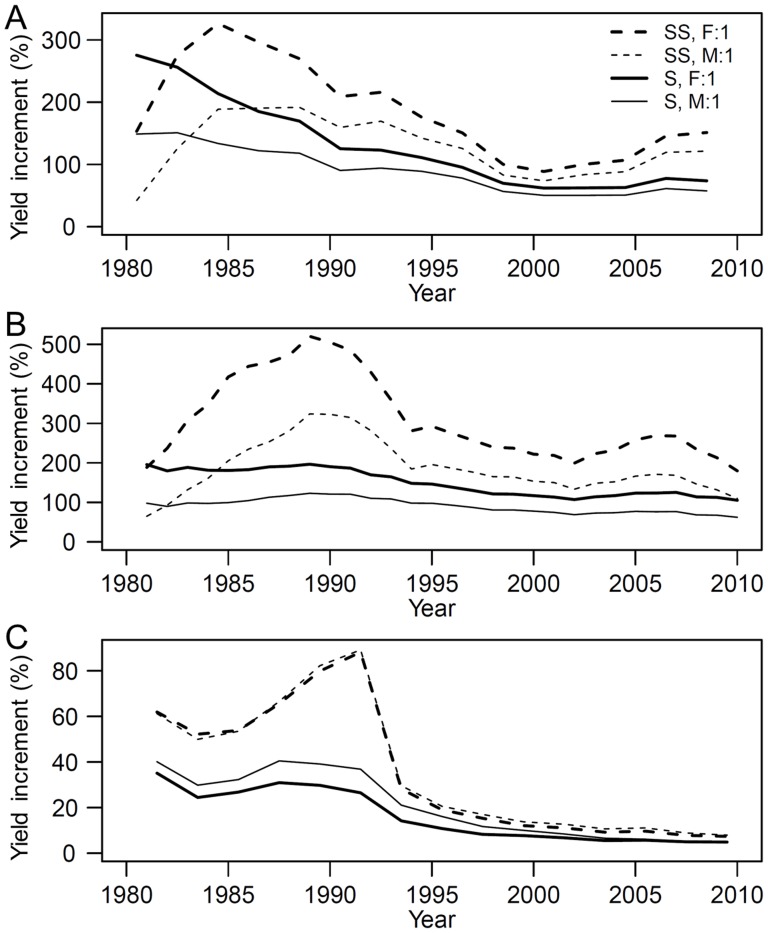
Temporal variations in the yield increment resulting from the application of fertilizer and farmyard manure. (A) Maize; (B) Barley; (C) Soybean; SS, subsurface soil; S, surface soil; F, level of fertilizer; M, level of farmyard manure.

In the sterile subsurface soil in the initial year, the application of fertilizer resulted in 153, 189, and 62% increases (the FYM resulted in 42, 65, and 61% increases) in crop yield for maize, barley, and soybean, respectively. In the fertile surface soil in the initial year, the application of fertilizer resulted in 275, 196, and 35% increases (FYM resulted in 149, 98, and 40% increases) in crop yield for maize, barley, and soybean, respectively. After 20 years of farming, the application of fertilizer resulted in 89, 219, and 11% increases (FYM resulted in 74, 150, and 13% increases) in the subsurface soil, and 62, 113, and 7% increases (FYM resulted in 50, 75, and 8% increases) in the surface soil for maize, barley, and soybean, respectively, because of the increased baseline fertility. The greatest yield increases were observed in the initial year in the fertile surface soil with the application of both fertilizer and FYM (compared with the treatment without fertilizer or FYM), and then the yield increment decreased each year (Fig. 4). In the sterile subsurface soil, it took 5–10 years for the yield increase to reach the maximum value.

### Predicted crop yields reflected the trend of the observed yields

The estimated fertility levels were used to predict the crop yield using the yield-fertility curves for each crop (Figs. S2A, S3A, and S4A). The trends of the predicted yield were similar to the trends of the observed yield for all three crops. The correlation coefficients were 0.758 (Fig. S2B, *P*<0.001) for maize, 0.801 (Fig. S3B, *P*<0.001) for barley, and 0.622 (Fig. S4B, *P*<0.001) for soybean.

Yield fluctuations were observed among the treatments but these fluctuations were not reflected by the predicted crop yields. For maize, the peak yields occurred in 1990 and 2004, and a reduced yield occurred in 1996. For barley, the peak yield for the fertilizer-treated plots occurred in 1993, but the peak yield occurred 1 year later in the plots without fertilizer treatment. The FYM treatments were discontinued in 1991 for the summer harvested crops (maize and soybean). Clear reductions in maize yield in 1991 and soybean yield in 1992 are shown in Fig. 1A and C, respectively, after which the yields for both crops increased until 2008. The estimated fertility levels and predicted yields represented the temporal variations that coincided with treatment changes during the experimental period (Fig. 1). Termination of the phosphate fertilizer application in 2001 was well described by the temporal variations in the baseline fertility of maize and barley. The baseline fertility levels declined, especially in the soil treated with FYM only (Fig. 4). Similar patterns were not observed for the fertilizer-treated soils. A decline in fertility was not observed for soybean. As previously mentioned, soybean yields were not significantly different among treatments.

### The effect of climatic factors

Crop yield can be affected by climatic factors [35] such as precipitation [44], [45], temperature [46], [47] and recent warming [48]. The impact of climate change may damage crop yields on a global scale [48], [49] and lead to decreases in crop production [50]. However, weather variation may play a small role in crop yield on a regional scale [45] and the effect may depend on fertilization and soil type [45], [50]. Monthly precipitation and average monthly temperature recorded at the experimental site are shown in Fig. S8A and B. We performed a correlation analysis between the residual of the crop yield (i.e., predicted yield minus observed yield) and the climatic variables for the three crops (Table S1 in File S1). The simple residual analysis indicated that temperature had some effect on maize and soybean yields. We then conducted additional analyses that included the effect of temperature in the crop yield-fertility model (see File S1 and Table S2 in File S1). Based on the results of the minor contribution of temperature, we concluded that the annual fluctuations in crop yields were mainly caused by unknown factors other than the climatic factors.

## Discussion

### The baseline fertility

The baseline fertility of the subsurface soil was very low for maize and barley. This suggests that the fertility in the subsurface soil prior to the application of fertilizer or FYM was not sufficient for maize or barley.

The increasing baseline fertility trend was also observed in soil without additional fertilizer or FYM. The increased fertility may have resulted from the addition of crop residues to the soil and nitrogen fixation by the soybean crop. These fertility increases may indicate soil maturation (i.e., the accumulation of nutrients) in the experimental fields.

The baseline fertility of maize indicated that when FYM was applied alone, soil fertility increased more than when both FYM and fertilizer were applied, for both soil types (Fig. 3A). The application of FYM increased the organic matter content of the soil, which resulted in increased soil fertility. Similar trends were observed for barley and soybean (Fig. 3B and C), supporting the results observed for maize. The results for soybean suggested that the application of fertilizer could decrease the soil fertility when soybean was planted in a soil with a saturated level of fertilization (Fig. 3C).

### The effects of fertilizer and FYM

Our result indicates that the amount of fertilizer applied in the experiment reached the fertility level at which the maize yield was half of the maximum yield, but the amount of FYM required to reach this value was much lower. Barley obtained the highest fertility from the soil and fertilization despite the fact that maize produced the greatest crop yield and had the largest half-saturated fertility level. By using nutrients from the soil and fertilizer, soybean was the crop that most often approached the maximum yield.

Our findings indicate that the sterile subsurface soil needed more time to accumulate nutrients than the surface soil. The percentage increments in crop yield were larger in the subsurface soil than in the surface soil, which indicates that fertilizer application effectively improved the crop yield in the barren field. The fertilizer-induced increment in maize and barley crop yield increased after 2001. This is because the phosphate fertilizer application was discontinued in 2001 and the baseline soil fertility subsequently declined. The soybean crop did not show a similar reduction in yield after 2001.

### Long-term field crop fertilization experiment and crop models

Long-term experiments examining fertilizer treatments and crop yield have been widely conducted to assess the effects of applying fertilizer [51], manure [52], or a combination of fertilizer and manure [14], [53], [54]. In addition, experiments have been conducted to determine the impacts of nutrient addition on the fertility status of different soils [55]–[57]. However, it is important to note that our long-term record of crop yields with various types of treatments has made this the first study to simultaneously examine the temporal variation in baseline fertility and the contribution of nutrient applications.

## Conclusions

By allowing the gradual temporal variation in baseline fertility, it was possible to estimate the process of soil fertilization and the short-term effects of different fertilizer treatments. Our results support the proposal that naturally derived nutrients should be used to maintain soil fertility and synthetic fertilizer should be used to maintain productivity. Our results provide a clear description of the relationship between crop yield and soil, which can be easily understood.

## Supporting Information

Figure S1 **Temporal variations in observed crop yield (g m^−2^) for six treatments in a field with a fertile surface soil and a field with a barren subsurface soil from 1980 to 2010.** (A) Maize; (B) Barley; (C) Soybean; SS, subsurface soil; S, surface soil; F, level of fertilizer; M, level of farmyard manure.
(TIF)

Figure S2 **Temporal variations in the observed and predicted yields (g m^−2^) of maize for six treatments in a field with a fertile surface soil and a field with a barren subsurface soil presented every second year from 1980 to 2008.** (A) A crop yield-fertility map. The crop yield-fertility model translates total fertility into predicted yield. The gray band represents the band that corresponds to the standard deviation (±SD) of the crop yield-fertility curve. (B) The relationship between the observed and predicted yields. SS, subsurface soil; S, surface soil; F, level of fertilizer; M, level of farmyard manure; BF, baseline fertility.
(TIF)

Figure S3 **As for Figure S2 but for barley every year from 1980 to 2010.**
(TIF)

Figure S4 **As for Figure S2 but for soybean every 2 years from 1981 to 2009.**
(TIF)

Figure S5 **Temporal variations in the baseline fertility estimated using the crop yield-fertility model for six treatments in a field with a fertile surface soil and a field with a barren subsurface soil from 1980 to 2010.** (A) Maize; (B) Barley; (C) Soybean; SS, subsurface soil; S, surface soil; F, level of fertilizer; M, level of farmyard manure.
(TIF)

Figure S6 **Temporal variations in the posterior means and the bands that correspond to the standard deviation (±SD) of the Bayesian estimates of baseline fertility for six treatments in a field with a fertile surface soil and a field with a barren subsurface soil.** (A) Maize; (B) Barley; (C) Soybean; SS, subsurface soil; S, surface soil; F, level of fertilizer; M, level of farmyard manure.
(TIF)

Figure S7 **Traces of the MCMC samples of** ***V*** **(g m^−2^),** ***K*** **,** ***a*** **, and** ***b*** **. (A) Maize; (B) Barley; (C) Soybean.** The chain length was set to 1,000,000 steps logging every 100^th^ step.
(TIF)

Figure S8 **Temporal variation of the climatic variables recorded at the experimental site from January 1980 to December 2011.** (A) Monthly precipitation. (B) Average monthly temperature.
(TIF)

File S1 **Contribution of the climatic factor.** The analyses by including a temperature effect in the crop yield-fertility model.
(DOCX)

Data S1 **The raw data of the crop yields of maize, barley, and soybean from the long-term fertilizer experiment from 1980 to 2010.** As for the detail of the experiment, see text.
(XLSX)
