# Supplementary figures and images for: Bayesian Inference of Baseline Fertility and Treatment Effects via a Crop Yield-Fertility Model
Source: PLoS One. 2014 Nov 18;9(11):e112785. doi: 10.1371/journal.pone.0112785 (PMC4236125; doi:10.1371/journal.pone.0112785)

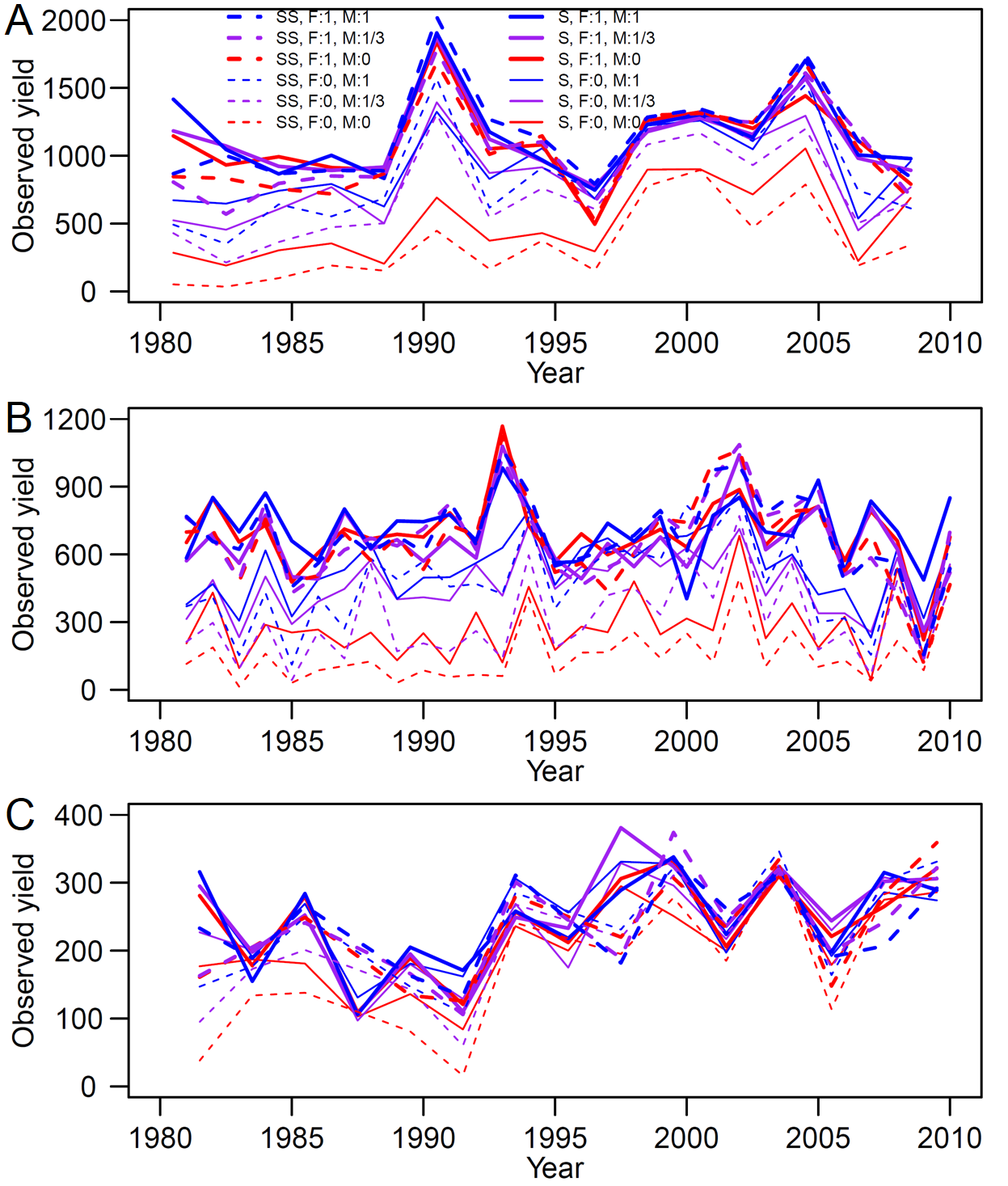

Supplement: Figure S1 — Temporal variations in observed crop yield (g m−2) for six treatments in a field with a fertile surface soil and a field with a barren subsurface soil from 1980 to 2010. (A) Maize; (B) Barley; (C) Soybean; SS, subsurface soil; S, surface soil; F, level of fertilizer; M, level of farmyard manure. (TIF) [file pone.0112785.s001.tif]

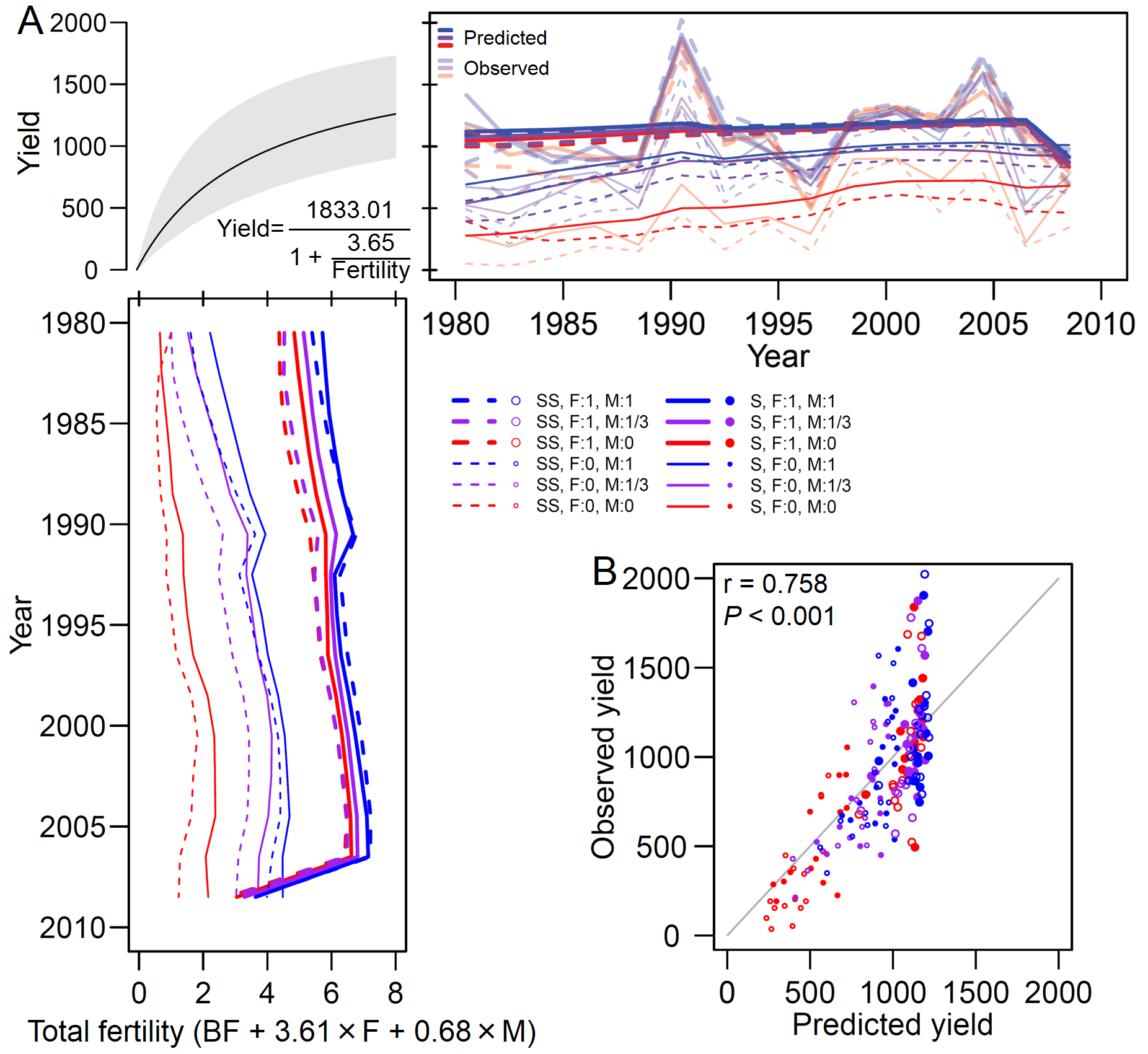

Supplement: Figure S2 — Temporal variations in the observed and predicted yields (g m−2) of maize for six treatments in a field with a fertile surface soil and a field with a barren subsurface soil presented every second year from 1980 to 2008. (A) A crop yield-fertility map. The crop yield-fertility model translates total fertility into predicted yield. The gray band represents the band that corresponds to the standard deviation (±SD) of the crop yield-fertility curve. (B) The relationship between the observed and predicted yields. SS, subsurface soil; S, surface soil; F, level of fertilizer; M, level of farmyard manure; BF, baseline fertility. (TIF) [file pone.0112785.s002.tif]

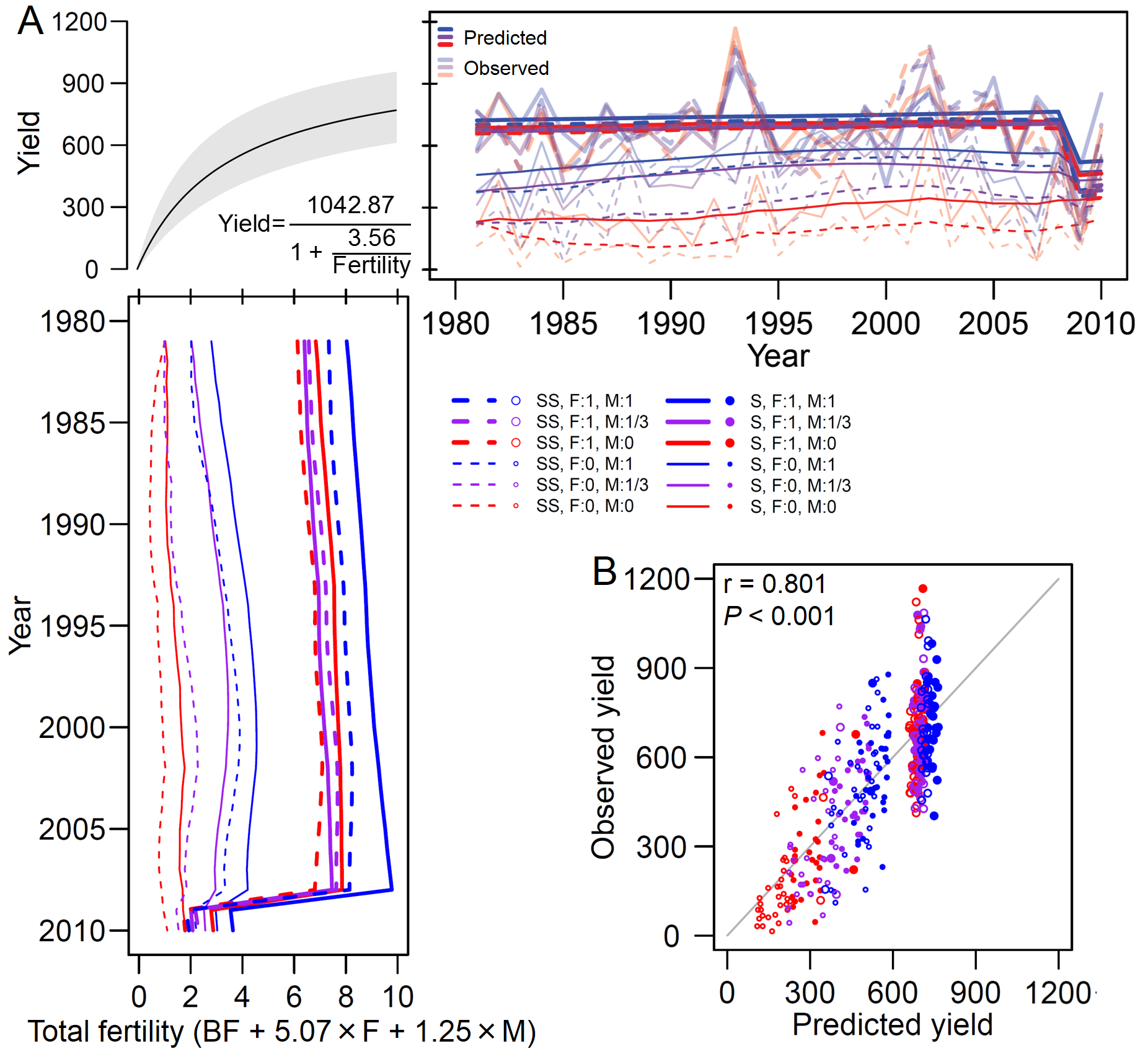

Supplement: Figure S3 — As for Figure S2 but for barley every year from 1980 to 2010. (TIF) [file pone.0112785.s003.tif]

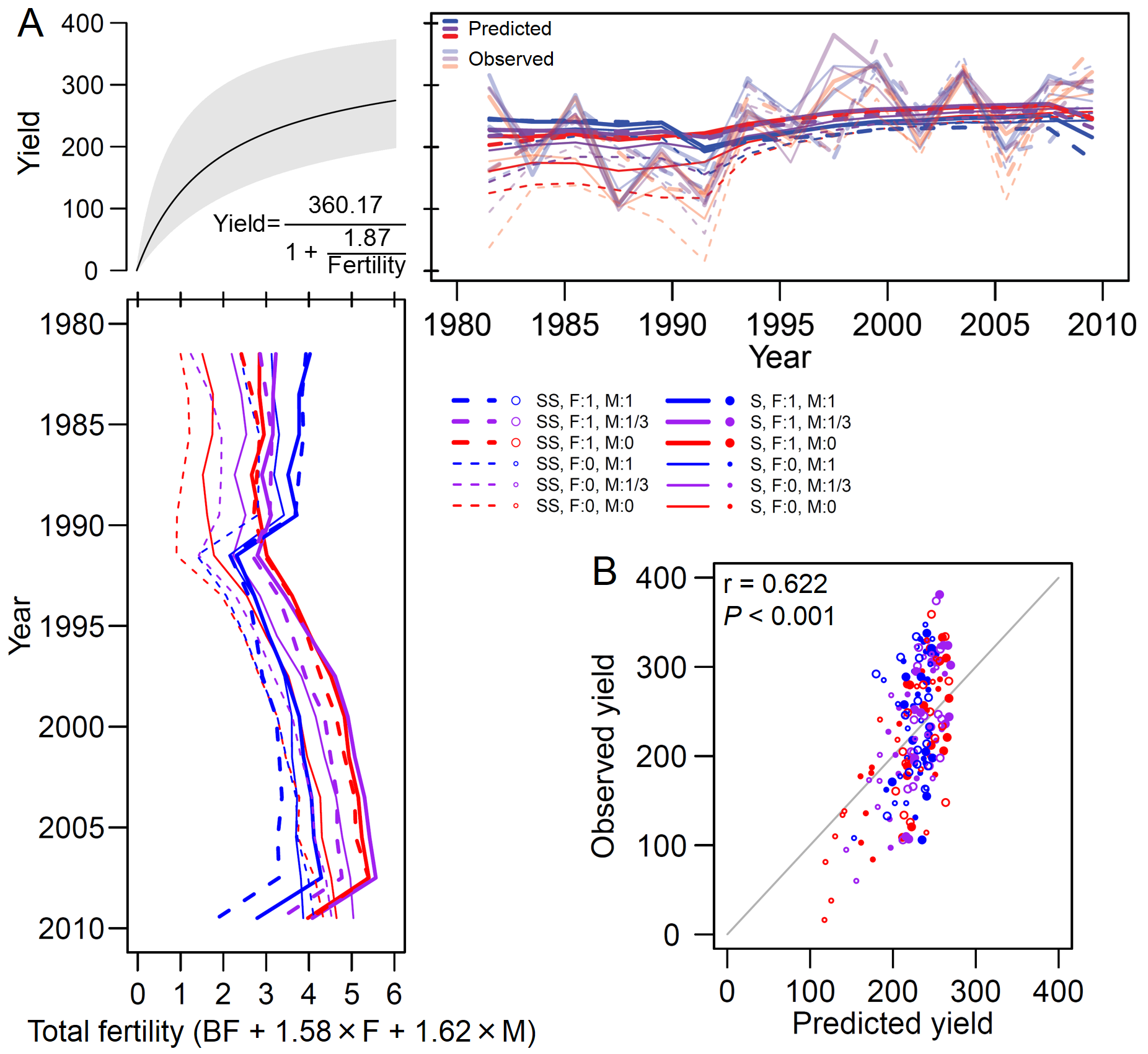

Supplement: Figure S4 — As for Figure S2 but for soybean every 2 years from 1981 to 2009. (TIF) [file pone.0112785.s004.tif]

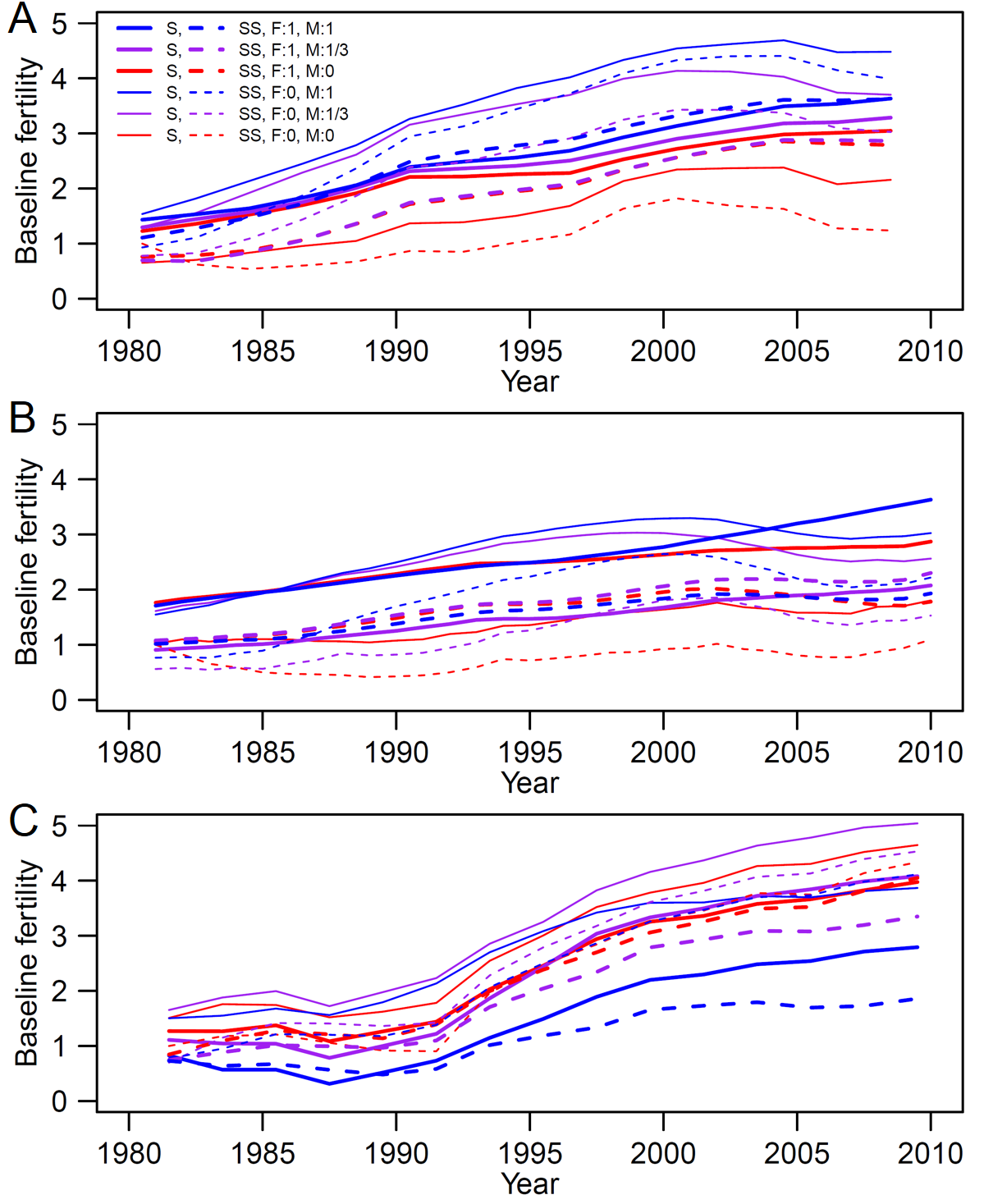

Supplement: Figure S5 — Temporal variations in the baseline fertility estimated using the crop yield-fertility model for six treatments in a field with a fertile surface soil and a field with a barren subsurface soil from 1980 to 2010. (A) Maize; (B) Barley; (C) Soybean; SS, subsurface soil; S, surface soil; F, level of fertilizer; M, level of farmyard manure. (TIF) [file pone.0112785.s005.tif]

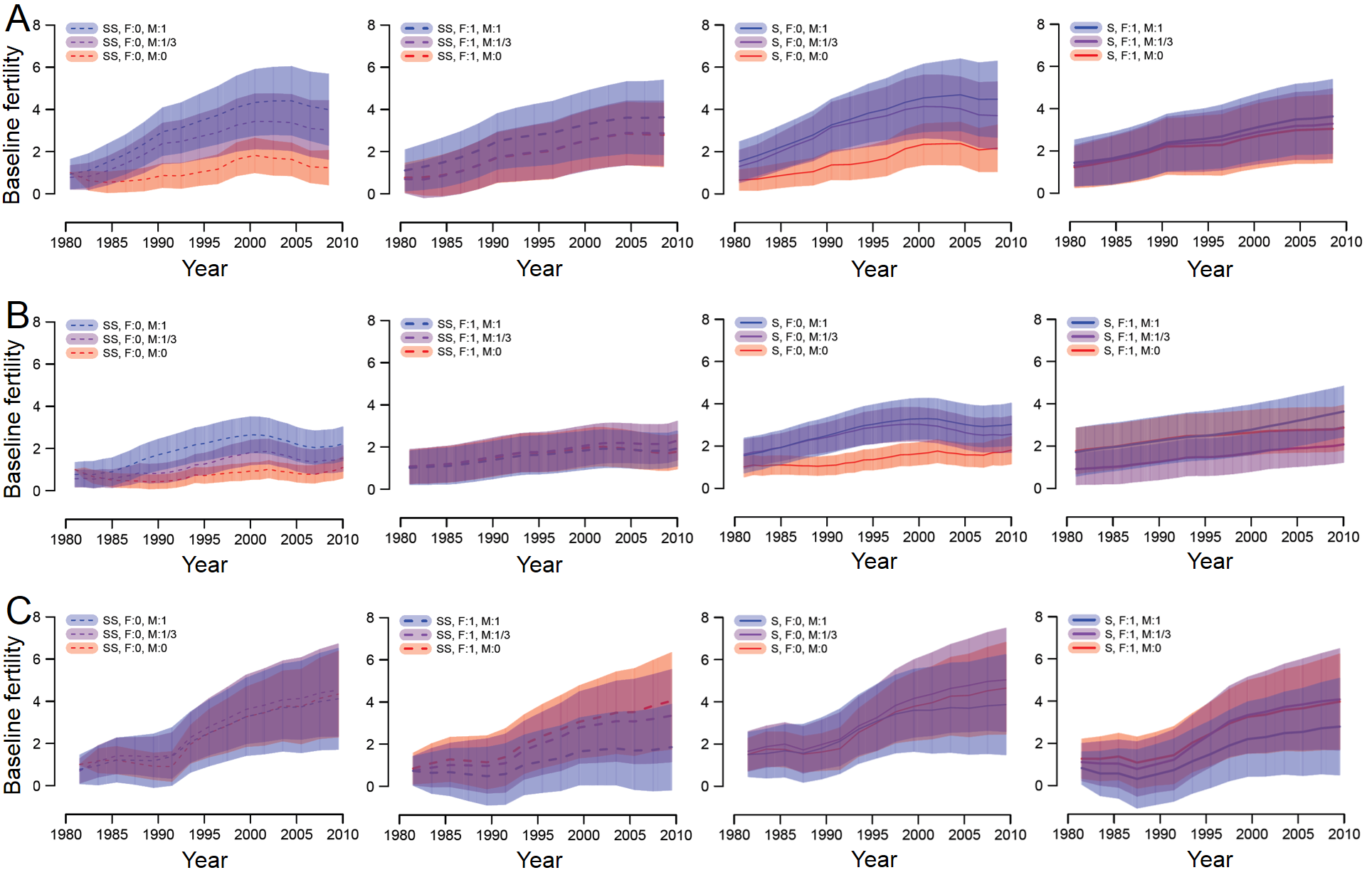

Supplement: Figure S6 — Temporal variations in the posterior means and the bands that correspond to the standard deviation (±SD) of the Bayesian estimates of baseline fertility for six treatments in a field with a fertile surface soil and a field with a barren subsurface soil. (A) Maize; (B) Barley; (C) Soybean; SS, subsurface soil; S, surface soil; F, level of fertilizer; M, level of farmyard manure. (TIF) [file pone.0112785.s006.tif]

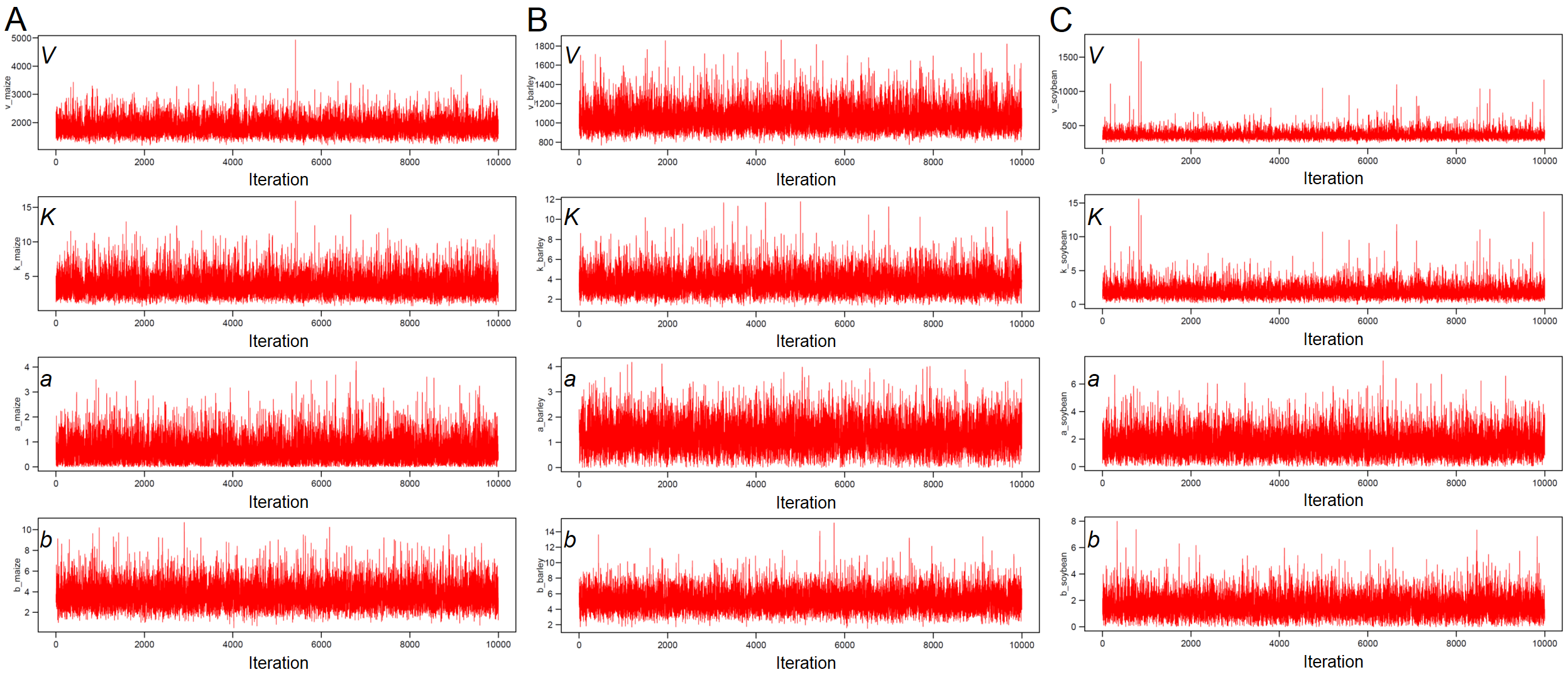

Supplement: Figure S7 — Traces of the MCMC samples of V (g m−2), K , a , and b . (A) Maize; (B) Barley; (C) Soybean. The chain length was set to 1,000,000 steps logging every 100th step. (TIF) [file pone.0112785.s007.tif]

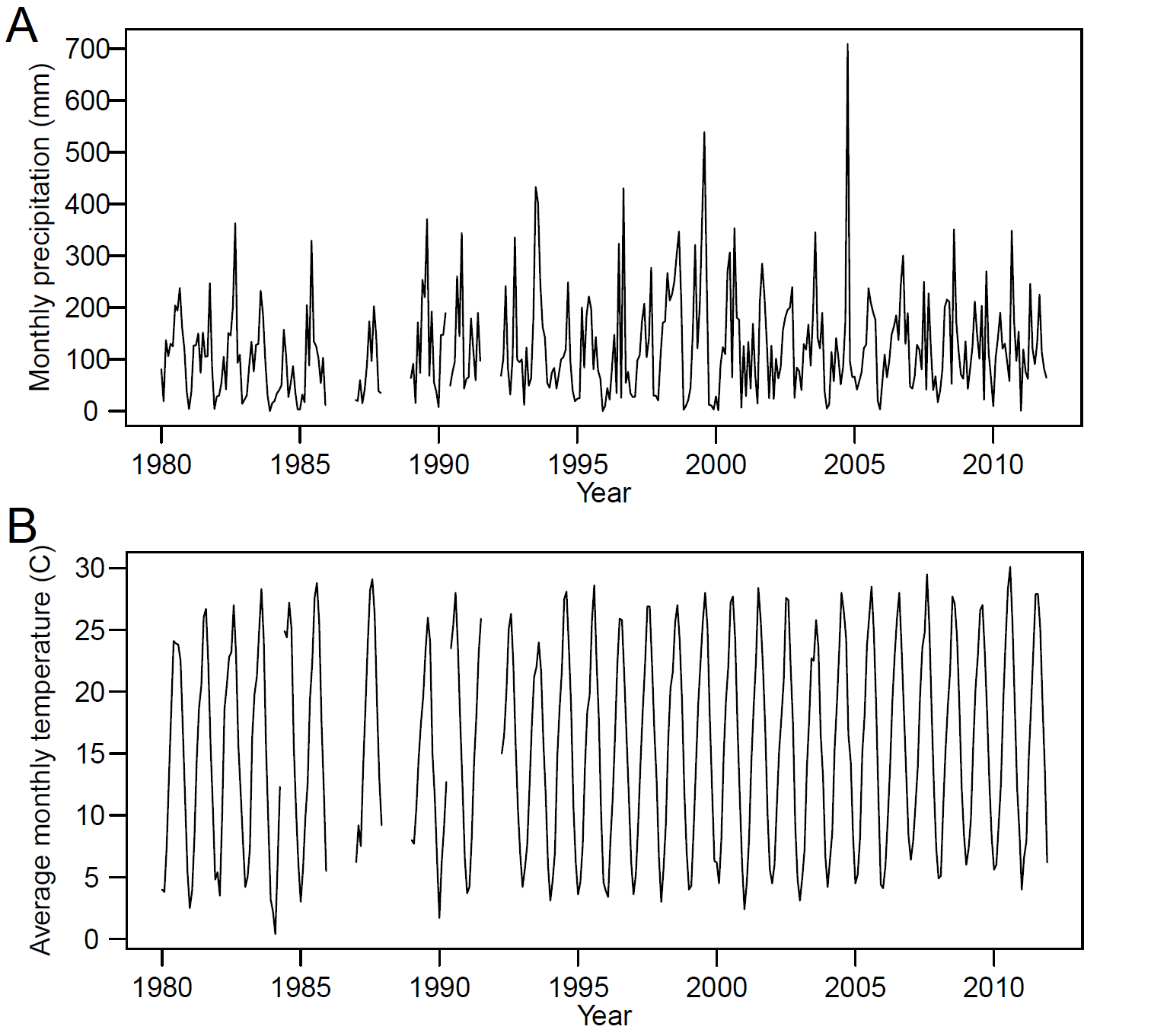

Supplement: Figure S8 — Temporal variation of the climatic variables recorded at the experimental site from January 1980 to December 2011. (A) Monthly precipitation. (B) Average monthly temperature. (TIF) [file pone.0112785.s008.tif]
