# Supplementary material for: Bayesian Inference of Baseline Fertility and Treatment Effects via a Crop Yield-Fertility Model
Source: PLoS One. 2014 Nov 18;9(11):e112785. doi: 10.1371/journal.pone.0112785 (PMC4236125; doi:10.1371/journal.pone.0112785)
Supplement: File S1 — Contribution of the climatic factor. The analyses by including a temperature effect in the crop yield-fertility model. (DOCX) [file pone.0112785.s009.docx]

**Supporting Information**

Bayesian Inference of Baseline Fertility and Treatment Effects via a Crop Yield-Fertility Model

Hungyen Chen^1^, Junko Yamagishi^2^, Hirohisa Kishino^1^

^1^Graduate School of Agricultural and Life Sciences, The University of Tokyo, Tokyo113-8657, Japan

^2^Institute for Sustainable Agro-ecosystem Services, The University of Tokyo, Tokyo 188-0002, Japan

Corresponding author: Hirohisa Kishino, 1-1-1 Yayoi, Bunkyo-ku, Tokyo 113-8657, Japan; Tel/Fax: +81-3-5841-5066; E-mail: kishino@lbm.ab.a.u-tokyo.ac.jp

**Contribution of the climatic factor**

We performed the analyses by including a temperature effect in the crop yield-fertility model. The model was modified as:

$Y_{i,t}\cong\frac{V}{1+\frac{K}{BF_{i,t}+a\times\text{F}+b\times\text{M}}+c\times\text{T}_{t}} .$ (S1)

The model relates crop yield (Y_i,t_) to the total fertility ($BF_{i,t}+a\times\text{F}+b\times\text{M}$) of the crop for treatment *i* at time *t*. *V* is the maximum yield at maximum fertilization; *K* is the fertility at which the yield is half of *V*; *BF_i,t_* is the baseline fertility for the crop for treatment *i* at time *t* and is assumed to vary gradually over time; F is the level of fertilizer; M is the level of the FYM; and *a* and *b* represent the contributions of F and M, respectively, relative to the baseline fertility. $c\times\text{T}_{t}$ represents the effect of temperature, and *c* represents the contribution of temperature (T) at time *t*. Hereafter, *V*, *K*, *a*, *b*, and *c* are referred to as the maximum yield, half-saturated fertility, fertilizer contribution, FYM contribution, and temperature contribution.

We conducted the analyses because the simple residual analysis implied minor temperature effects on maize and soybean (Table S1). The results of the Bayesian estimates of the modified crop yield-fertility model for the three crops are shown in Table S2. The temperature contribution to the maize yield was 99.7 and 98.6% smaller than the fertilizer and the FYM contribution, respectively. The temperature contribution to the barley yield was 99.0 and 95.8% smaller than the fertilizer and the FYM contribution, respectively, and 89.3 and 81.4% smaller than the fertilizer and the FYM contribution, respectively, to the soybean yield. These results indicate that the effect of temperature was considerably smaller than the effect of the fertilizer and the FYM on the crops examined in this study.

Table S1 Results of the correlation analysis between the residual of the crop yield and the climatic variables.

|  | Precipitation | | Temperature | |
| --- | --- | --- | --- | --- |
|  | Correlation coefficient | *P*-value | Correlation coefficient | *P*-value |
| Maize |  |  |  |  |
| Surface soil |  |  |  |  |
| F:0, M:0 | 0.032 | 0.91 | -0.321 | 0.28 |
| F:0, M:1/3 | 0.095 | 0.76 | -0.295 | 0.33 |
| F:0, M:1 | 0.160 | 0.60 | -0.397 | 0.18 |
| F:1, M:0 | 0.235 | 0.44 | -0.336 | 0.26 |
| F:1, M:1/3 | 0.210 | 0.49 | -0.175 | 0.57 |
| F:1, M:1 | 0.182 | 0.55 | -0.106 | 0.73 |
| Subsurface soil |  |  |  |  |
| F:0, M:0 | -0.042 | 0.89 | -0.666 | 0.01 |
| F:0, M:1/3 | -0.016 | 0.96 | -0.309 | 0.30 |
| F:0, M:1 | 0.088 | 0.77 | -0.384 | 0.20 |
| F:1, M:0 | 0.209 | 0.49 | -0.510 | 0.07 |
| F:1, M:1/3 | 0.295 | 0.33 | -0.543 | 0.06 |
| F:1, M:1 | 0.277 | 0.36 | -0.407 | 0.17 |
| Barley |  |  |  |  |
| Surface soil |  |  |  |  |
| F:0, M:0 | 0.204 | 0.30 | -0.002 | 0.99 |
| F:0, M:1/3 | 0.297 | 0.13 | 0.053 | 0.79 |
| F:0, M:1 | 0.200 | 0.31 | 0.226 | 0.25 |
| F:1, M:0 | -0.081 | 0.68 | 0.094 | 0.63 |
| F:1, M:1/3 | -0.053 | 0.79 | 0.066 | 0.74 |
| F:1, M:1 | -0.176 | 0.37 | 0.046 | 0.81 |
| Subsurface soil |  |  |  |  |
| F:0, M:0 | 0.112 | 0.57 | 0.079 | 0.69 |
| F:0, M:1/3 | 0.232 | 0.23 | 0.063 | 0.75 |
| F:0, M:1 | 0.031 | 0.87 | 0.120 | 0.54 |
| F:1, M:0 | -0.072 | 0.72 | 0.102 | 0.60 |
| F:1, M:1/3 | -0.162 | 0.41 | 0.192 | 0.33 |
| F:1, M:1 | -0.183 | 0.35 | 0.228 | 0.24 |
| Soybean |  |  |  |  |
| Surface soil |  |  |  |  |
| F:0, M:0 | -0.206 | 0.46 | 0.575 | 0.03 |
| F:0, M:1/3 | -0.280 | 0.31 | 0.476 | 0.07 |
| F:0, M:1 | -0.279 | 0.31 | 0.439 | 0.10 |
| F:1, M:0 | -0.236 | 0.40 | 0.407 | 0.13 |
| F:1, M:1/3 | -0.187 | 0.50 | 0.449 | 0.09 |
| F:1, M:1 | -0.225 | 0.42 | 0.327 | 0.23 |
| Subsurface soil |  |  |  |  |
| F:0, M:0 | -0.357 | 0.19 | 0.366 | 0.18 |
| F:0, M:1/3 | -0.450 | 0.09 | 0.366 | 0.18 |
| F:0, M:1 | -0.343 | 0.21 | 0.254 | 0.36 |
| F:1, M:0 | -0.215 | 0.44 | 0.255 | 0.36 |
| F:1, M:1/3 | -0.552 | 0.03 | 0.295 | 0.29 |
| F:1, M:1 | -0.476 | 0.07 | 0.309 | 0.26 |

Residual, predicted yield minus observed yield. Precipitation, mean of the monthly precipitation in the growing season for maize (July–September), barley (November, December, January–May), and soybean (July–October); Temperature, mean of the average monthly temperature in the growing season; F, level of fertilizer; M, level of farmyard manure.

Table S2 The posterior mean and standard deviation (SD) of the Bayesian estimates.

| Crop | *V* (g m^-2^) | | *K* | | *a* | | *b* | | *c* | |
| --- | --- | --- | --- | --- | --- | --- | --- | --- | --- | --- |
|  | Mean | SD | Mean | SD | Mean | SD | Mean | SD | Mean | SD |
| Maize | 1887.39 | 377.15 | 3.96 | 1.79 | 3.55 | 1.24 | 0.71 | 0.54 | 0.01 | 0.01 |
| Barley | 1064.81 | 152.37 | 3.67 | 1.23 | 4.84 | 1.37 | 1.20 | 0.61 | 0.05 | 0.03 |
| Soybean | 341.06 | 38.81 | 1.60 | 0.65 | 1.21 | 0.72 | 0.70 | 0.56 | 0.13 | 0.03 |

*V*, maximum yield; *K*, half-saturated fertility; *a*, fertilizer contribution; *b*, FYM contribution; *c*, temperature contribution.
